# Impressions of a Russian Hospital

**Published:** 1897-09-25

**Authors:** 


### IMPRESSIONS OF A RUSSIAN HOSPITAL.

By a Nurse Correspondent.

Making a short stay in St. Petersburg and Moscow early this winter, and having already seen the principal institutions in these cities of palaces and churches, I was fortunate enough, through the kindly intervention of the Rev. Alex Francis, minister to the Congregational Church in St. Petersburg, to obtain the necessary permission to go over a Russian hospital. Several English doctors, another nurse and myself formed a party for the occasion, and our way was made smooth before us.

The visit promised something of interest and novelty, though our medical *confrères* seemed to airily assume that a Russian hospital, even in a comparatively new city like St. Petersburg, would carry us back to mediævalism, and that the treatment of the patients would be—to put it civilly—pre-scientific.

Before we had finished, I think we were quite willing to acknowledge this to be an error. All, I should say, but one medical student from Guy's, who remained disdainful of methods not closely following those in his own training school, and to the end refused to be reconciled to things Russian.

The hospital chosen for our inspection was typical and characteristic, but the first thing that struck us on arriving was the absence of the architectural grandeur that marks most public buildings in St. Petersburg. We were taken through the out-patient department, which was clean and bare, and much like other such quarters at home, though somewhat lacking in comfort. Indeed, Russians of the class expected in hospital seem to have little notion of comfort, unless one allows that warmth through overcrowding is a factor in comfort. As we passed through the secretarial department I noticed that some of the clerks were ladies. The senior physician to the hospital—a German of high scientific reputation—received us with courtesy, and escorting us through the wards, introduced us to the surgeons in their department. In this, and I understand the practice obtains in other Russian hospitals, they adopt the system of special wards for special cases. The medical wards were filled —over-filled if one measured the floor-space—with patients. Enteric fever cases in large wards, in small wards, and in corridor wards were present in great numbers. Cases of renal disease seemed to come next in number. In separate wards patients with lung trouble—pneumonia and pleurisy —were treated. So far as I could learn no cases of phthisis were included.

A physician's chart diagnosing and prescribing hung by the side of each bed, and, being written in Latin, was possible to read—though the apothecaries' weight was perplexing, but the neighbouring chart in Russian caligraphy might have been Choctau for all we could gather from it. We then visited the basement where the delirium tremens cases were nursed. In proportion their number seemed large. Russian Vodkà, it is said, is a most villainous form of brandy, and works more terrible havoc amongst its consumers than does the beer or gin dispensed to the English working classes. These poor people looked dismal and cadaverous; they were quiet, most of them, however, being restrained by having long strips of sheeting folded crosswise over the chest and fastened to each side of the bed. Male nurses, tall and strong, wearing white uniforms, had charge of these patients, and though some of the refinements of English nursing were absent, the attendants appeared kind and capable.

We then visited the surgical wards and the operating theatre, where the arrangements were modelled after German methods. Indeed, several of the senior surgeons were of German nationality. Two cases of trephining for Jacksonian epilepsy and a plastic operation were the triumphs of the hour, and the surgeon was warmly congratulated on his success by the English doctors.

In one of the corridors we encountered a Russian priest, a fine-looking man, but who offended one's taste by wearing a long, thick beard, and bushy hair, artificially crimped, hanging over his shoulders. We returned his bow as he passed us—we were too English to take the initiative in saluting, but Russians invariably bow when passing a church, a holy image, or a priest.

After this we had just time to skim through a large circular hospital built after a more modern design and fitted with more modern appliances, but which to English visitors was perhaps of less interest on that account. One or two of us also looked over the chapel attached and admired the Icons or holy images, without which no house, no room in any Russian dwelling is considered complete for Christian habitation.

After a flying visit such as this it would be hardly fair to attempt an opinion of the nursing in Russian hospitals, nor perhaps of the nurses. I admired a handsome girl who was performing massage, and the chief nurse in the theatre, who was alert and intelligent to her finger-tips; but the casual English visitor would not hesitate to say that the Russian nurses do not come up to our standard at home. But the same loyal Briton would certainly say that the Russian women who are not nurses fall equally short of our standard at home.

Contrary to my expectation, there was no sign of smoking nor scent of tobacco throughout the hospitals.

In this hospital the nurses wore uniform and badges on the arm; they appeared bright and happy, and seemed to be on professionally fraternal terms with the clinical clerks and dressers, with whom they worked, and they were not fretted with the presence of a stern sister to chaperone them. The young men were garbed in white operation coats and aprons, put on very neatly, wherein they differed from their seniors, who were somewhat careless in the matter of tapes and so on—one distinguished man wearing an apron that trailed an inch and a half on the ground, though he never once tripped over it as a mere woman might have done.

The nurses' dresses made a refreshing spot in a hospital where æsthetic taste is not regarded. It almost jarred on one to see the mud-coloured blankets and clay-coloured dressing-gowns and bed-jackets used by these sallow-skinned Russian patients. The same materials in red, in scarlet and grey, or in orange-colour would have cost little more, and would have brightened up their naturally dull complexions and lent a warm note of colour to the ward. That, however, is a detail too trivial to mar the gratification our visit to the hospitals gave us, and our satisfaction in learning that even in unchanging Russia there is increasing care and devotion given to the sick.

